# Relative Contributions of Quinolone Resistance-Determining Region Mutations and Efflux Pump-Associated Mechanisms to Fluoroquinolone Resistance in *Staphylococcus aureus* Clinical Isolates and Their Associations with Virulence Gene Profiles

**DOI:** 10.3390/antibiotics15101006

**Published:** 2026-10-10

**Authors:** Sohyeong Kim, Heechul Park, Minju Jung, Soyun Kim, Jieun Lim, Choseung Kim, Sangha Kim, Jinyoung Bae, Jiyoung Lee, Min Park, Sunghyun Kim

**Affiliations:** 1 Department of Clinical Laboratory Science, College of Health Sciences, Catholic University of Pusan, Busan 46252, Republic of Korea; hyeongso29@naver.com (S.K.); jungm9911@naver.com (M.J.); b116398@naver.com (S.K.); ieun0418@naver.com (J.L.); 2 Next-Generation Industrial Field-Based Specialist Program for Molecular Diagnostics, Brain Busan 21 Plus Project, Graduate School, Catholic University of Pusan, Busan 46252, Republic of Korea; ea0925@naver.com; 3 Department of Clinical Laboratory Science, Hyejeon University, Hongseong 32244, Republic of Korea; phc2626@hj.ac.kr; 4 Department of Forensic Science, Graduate School, Catholic University of Pusan, Busan 46252, Republic of Korea; 5 Department of Laboratory Medicine, Konyang University Hospital, Daejeon 35365, Republic of Korea; comi0110@hanmail.net; 6 Department of Research & Development, Q-Solutions, Changwon 51508, Republic of Korea; bjyscene@kims.re.kr; 7 Division of Research & Development, Dream Dx Inc., Busan 48059, Republic of Korea; lab84@dreamdx.net; 8 Department of Biomedical Laboratory Science, Masan University, Changwon 51217, Republic of Korea

**Keywords:** *Staphylococcus aureus*, fluoroquinolone resistance, ciprofloxacin, levofloxacin, carbonyl cyanide 3-chlorophenylhydrazone (CCCP), multidrug resistance, efflux pump, quinolone resistance-determining region (QRDR), antimicrobial susceptibility, virulence factors

## Abstract

**Background**: Fluoroquinolone resistance in methicillin-resistant *Staphylococcus aureus* (MRSA) involves multiple mechanisms, including quinolone resistance-determining region (QRDR) mutations and efflux pump activity. However, the relationship between these resistance mechanisms and virulence factors (VFs) remains unclear. This study investigated the relative contributions of QRDR mutations and carbonyl cyanide 3-chlorophenylhydrazone (CCCP)-responsive mechanisms to fluoroquinolone resistance in MRSA and their associations with VF profiles. **Methods**: Sixty ciprofloxacin-resistant MRSA bloodstream isolates were analyzed. The minimum inhibitory concentrations (MICs) of ciprofloxacin (CIP) and levofloxacin (LEV) were determined before and after treatment with the efflux pump inhibitor CCCP. The isolates were classified as CCCP responsive or non-responsive based on a ≥4-fold reduction in CIP MICs. The associations of QRDR mutations in *gyr*A, *gyr*B, *par*C, and *par*E and virulence-associated genes with CCCP responsiveness were evaluated. **Results**: CCCP significantly reduced both CIP and LEV MICs, although changes in LEV MICs were limited to a subset of isolates. Of the 60 isolates, 15 were CCCP responsive, whereas 45 were non-responsive. The non-responsive isolates had significantly more QRDR mutations, particularly *gyr*A nucleotide mutations and GyrA amino acid substitutions. *fnbA*-positive isolates showed greater CCCP-mediated CIP MIC reductions and fewer QRDR nucleotide mutations, whereas *seg*-, *sei*-, and *tst*-positive isolates showed lower CCCP-mediated CIP MIC reductions and greater QRDR nucleotide mutation numbers. **Conclusions**: QRDR mutations and CCCP-responsive mechanisms were differentially associated with fluoroquinolone resistance phenotypes in MRSA. Their associations with distinct VF profiles suggest a potential relationship between CCCP responsiveness, QRDR mutation profiles, and the virulence genetic background of MRSA.

## 1. Introduction

Methicillin-resistant *Staphylococcus aureus* (MRSA) is characterized by resistance to methicillin and other β-lactam antibiotics and is responsible for a broad spectrum of infections, including severe invasive and bloodstream infections. Moreover, its resistance to multiple antimicrobial classes restricts available therapeutic options [1]. Reflecting this continuing burden, antibiotic-resistant *S. aureus*, including MRSA, remains a high-priority pathogen on the 2024 World Health Organization Bacterial Priority Pathogens List [2]. Fluoroquinolone resistance common in MRSA isolates further complicates the management of multidrug-resistant *S. aureus* [3].

Fluoroquinolones exert their antibacterial activity primarily by targeting DNA gyrase and topoisomerase IV, which are essential for bacterial DNA replication. Fluoroquinolone resistance in *S. aureus* is primarily associated with mutations within the quinolone resistance-determining regions (QRDRs) of *gyr*A and *gyr*B, which encode DNA gyrase, and *par*C and *par*E, which encode topoisomerase IV [3]. Accumulation of target site mutations can progressively reduce fluoroquinolone susceptibility and contribute to high-level resistance. In our previous study, we analyzed fluoroquinolone-resistant *S. aureus* clinical isolates and demonstrated that QRDR mutations, particularly specific amino acid substitutions in *parC*, are associated with high fluoroquinolone minimum inhibitory concentrations (MICs) and that combined substitutions at positions S80 and E84 are associated with high resistance levels [4].

However, target site mutations are not the only mechanisms contributing to fluoroquinolone resistance in *S. aureus*. Active drug efflux pumps mediated by multidrug efflux pumps can decrease intracellular antimicrobial concentrations, thereby reducing fluoroquinolone susceptibility [5]. Several chromosomally encoded efflux pump systems, including *nor*A, *nor*B, and *nor*C, have been implicated in fluoroquinolone resistance, with *nor*A being one of the most extensively characterized systems and showing activity, particularly against hydrophilic fluoroquinolones such as ciprofloxacin (CIP) [6]. In addition to the Nor family of transporters, other chromosomally encoded multidrug efflux systems, including MepA and MdeA, have been described in *S. aureus* [7]. Importantly, efflux-mediated resistance may coexist with QRDR target alterations, suggesting that the relative contributions of these mechanisms vary among clinical isolates. Therefore, phenotypic assessments using efflux pump inhibitors should be conducted to clarify the contribution of active efflux to fluoroquinolone resistance [8,9].

Carbonyl cyanide 3-chlorophenylhydrazone (CCCP), a protonophore that disrupts the proton motive force, has been widely used as an efflux pump inhibitor to investigate the contribution of active efflux pumps to antimicrobial resistance in Gram-positive (e.g., *S. aureus*) and Gram-negative bacteria [10,11,12,13].

In addition to antimicrobial resistance, the pathogenicity of *S. aureus* is determined by different virulence factors (VFs) that contribute to bacterial adhesion, tissue damage, immune dysregulation, and disease development. Adhesion-associated factors, including clumping factors (*clf*A and *clf*B) and fibronectin-binding proteins (*fnb*A and *fnb*B), promote bacterial attachment to host tissues and extracellular matrix components, and facilitate colonization [14]. Hemolysins, including alpha-, beta-, gamma-, and delta-hemolysins, contribute to host cell damage and tissue injury [15]. Superantigenic toxins, such as toxic shock syndrome toxin-1 and staphylococcal enterotoxins, can induce excessive and dysregulated immune activation, contributing to toxic shock syndrome and foodborne disease [16]. Other toxin-associated VFs, including Panton–Valentine leukocidin (PVL), LukED, and exfoliative toxins, contribute to leukocyte injury and tissue damage and have been implicated in a range of skin and invasive infections. [17]. Collectively, these VFs contribute to the ability of *S. aureus* to colonize, invade, and evade host defenses and cause a broad spectrum of infections [18].

The relationship between antimicrobial resistance and virulence in *S. aureus* is complex. Previous studies have investigated antimicrobial resistance and virulence characteristics within the same clinical isolate population. Thompson and Brown compared resistance phenotypes to 11 anti-staphylococcal antibiotics, the presence of 35 virulence genes, and pathogenicity between clinical MRSA and methicillin-susceptible *S. aureus* (MSSA) isolates and demonstrated that resistance, virulence gene repertoire, and pathogenicity do not necessarily vary in parallel [19]. In addition, multidrug efflux systems have been linked to phenotypes other than antimicrobial resistance. Pharmacological inhibition of the *nor*A efflux pump reportedly enhances CIP activity and reduces biofilm formation in *S. aureus* [20]. Similarly, inhibition of the NorA-mediated efflux pump by boeravinone B reduces CIP MIC, biofilm formation, and intracellular invasion, further indicating an association between efflux pump-related phenotypes and virulence-associated characteristics [21].

These studies have primarily addressed antimicrobial resistance phenotypes, virulence gene repertoires, or specific virulence-associated phenotypes such as biofilm formation and intracellular invasion. Although QRDR target site mutations and multidrug efflux pump systems are established contributors to fluoroquinolone resistance in *S. aureus*, the relationship between these resistance mechanisms remains insufficiently characterized. In particular, whether QRDR mutation accumulation and efflux pump-mediated resistance contribute independently, concurrently, or alternatively to fluoroquinolone resistance in clinical MRSA isolates has not been established. Moreover, the relationships between individual virulence gene profiles and the distinct components of fluoroquinolone resistance remain poorly understood. Thus, whether particular virulence gene profiles are associated with the different relative contributions of QRDR mutations and efflux pump activation to fluoroquinolone resistance remains unclear.

Therefore, the present study aimed to investigate the relative contributions of QRDR mutations and CCCP-responsive changes in fluoroquinolone susceptibility in clinical MRSA isolates and to determine whether these resistance phenotypes are associated with distinct virulence gene profiles. Changes in CIP and levofloxacin (LEV) MICs after CCCP treatment were evaluated, and virulence-associated genes were characterized using SYBR Green-based quantitative real-time PCR (qPCR). The results of these analyses were integrated with previously determined QRDR mutation profiles.

## 2. Results

### 2.1. Selection of the Working Concentration of CCCP

The effect of different CCCP concentrations (0.1–0.2 μg/mL) on bacterial growth was evaluated by measuring OD_600_. At 0.1 μg/mL CCCP, six of the eight tested *S. aureus* strains maintained OD_600_ values ≥ 50% of their respective untreated controls, and the mean relative OD_600_ across all eight strains was approximately 52.7%. Higher CCCP concentrations resulted in greater overall reductions in OD_600_ on average. Therefore, 0.1 μg/mL CCCP was selected as the working concentration for subsequent efflux inhibition and antimicrobial susceptibility experiments.

### 2.2. Effect of CCCP on Fluoroquinolone MICs and Classification of MRSA Isolates

**Figure 1:**
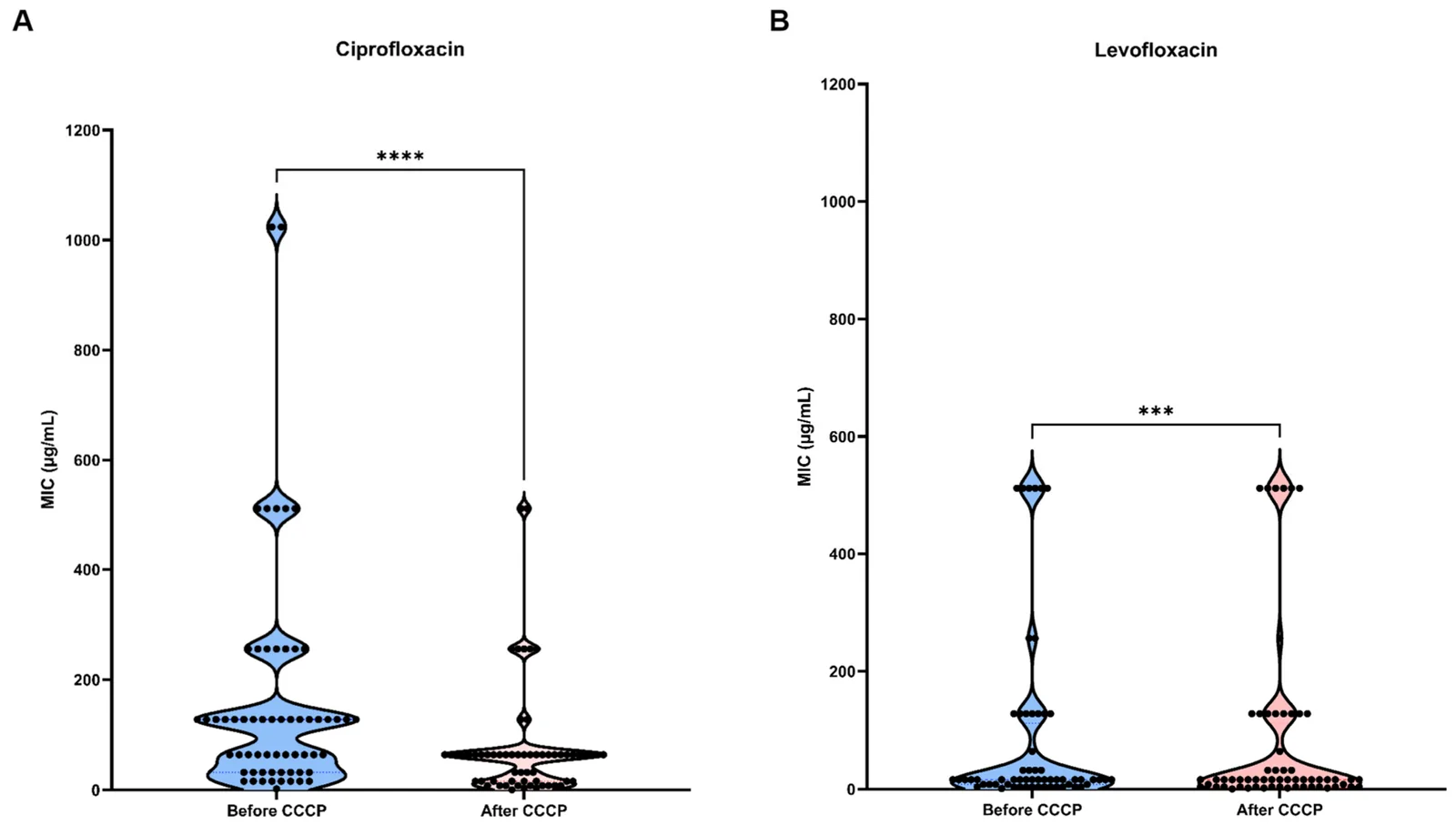
Comparison of fluoroquinolone MICs before and after CCCP treatment. Violin plots showing the distributions of (**A**) ciprofloxacin (CIP) and (**B**) levofloxacin (LEV) MICs before and after treatment with CCCP (0.1 μg/mL) in 60 MRSA isolates. Individual dots represent individual isolates, and dotted lines indicate the median and interquartile range. MICs before and after CCCP treatment were compared using the Wilcoxon matched-pairs signed-rank test. *** *p* < 0.001 and **** *p* < 0.0001; ns, not significant.

The effect of CCCP on fluoroquinolone susceptibility was evaluated in 60 MRSA isolates by comparing the MICs of CIP and LEV before and after CCCP treatment. CCCP treatment significantly reduced the MICs of both CIP and LEV (CIP, *p* < 0.0001; LEV, *p* = 0.0005) (Figure 1). However, changes in LEV MICs were limited to a subset of isolates, with 48 of the 60 isolates showing no change after CCCP treatment.

Based on the predefined criterion of a ≥4-fold reduction in CIP MICs, 15 of the 60 isolates (25.0%) were classified into the CCCP-responsive group, whereas 45 isolates (75.0%) showing a <4-fold reduction were classified into the CCCP-non-responsive group. The isolate-specific MICs of CIP and LEV before and after CCCP treatment are presented in Table 1.

### 2.3. Association Between CCCP Responsiveness and QRDR Mutation Numbers

**Figure 2:**
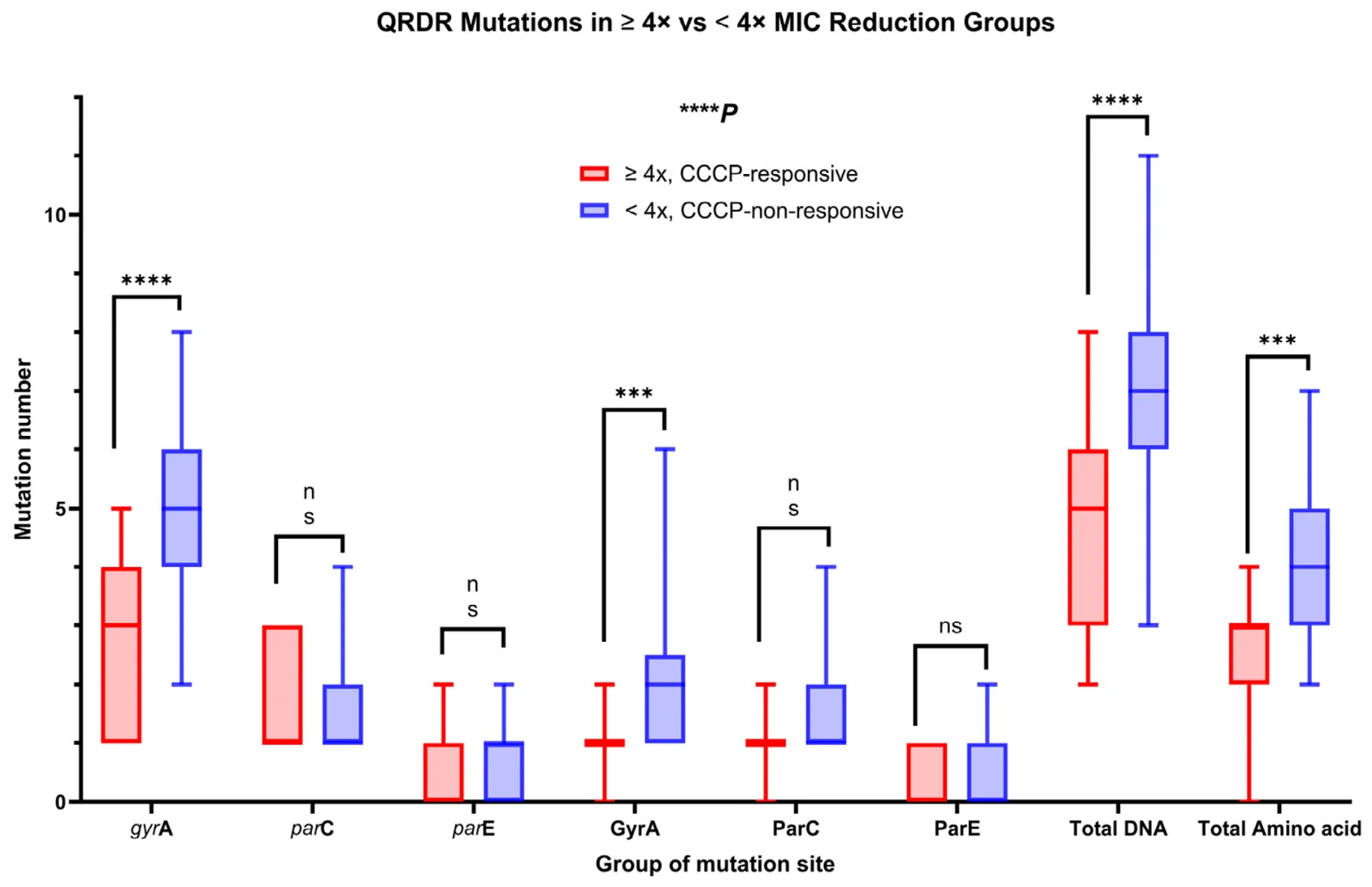
Comparison of QRDR mutation numbers between CIP-based CCCP-responsive (≥4-fold reduction in CIP MIC) and CCCP-non-responsive (<4-fold reduction in CIP MIC) groups. *** *p* < 0.001 and **** *p* < 0.0001; ns, not significant.

Previously characterized QRDR mutation profiles were reanalyzed according to CCCP responsiveness, which was defined based on a ≥4-fold reduction in CIP MIC. Overall, the number of QRDR mutations significantly differed between the CCCP-responsive (≥4-fold) and CCCP-non-responsive (<4-fold) groups (*p* < 0.0001; Figure 2). At the individual gene level, the CCCP-non-responsive group exhibited significantly more *gyr*A nucleotide mutations and GyrA amino acid substitutions than the CCCP-responsive group (*p* < 0.0001 and *p* = 0.0003, respectively). No significant differences between the two groups were observed for *par*C/ParC or *par*E/ParE. No mutations were identified in *gyr*B among the 60 MRSA isolates.

When QRDR mutations were considered collectively, the CCCP-non-responsive group showed significantly more total nucleotide mutations and amino acid substitutions than the CCCP-responsive group (*p* < 0.0001 and *p* = 0.0004, respectively).

### 2.4. Distribution of VF Genes Among MRSA Isolates

Among the 21 VF genes examined, *clf*A, *clf*B, *hla*, *hlb*, *hld*, and *lukED* were detected in all isolates, whereas *hlg*, *sea*, *seb*, *see*, *seh*, *eta*, and *etb* were not detected in any isolate. As these genes showed no variation in prevalence among the isolates, they were excluded from subsequent comparative analyses. Therefore, statistical comparisons between the CCCP-responsive and -non-responsive groups were performed using the remaining eight VF genes that exhibited variable expression among the isolates. The distribution of the eight genes with variable prevalence is summarized in Table 2.

The prevalence of the eight VF genes showing variable distributions was compared between the CCCP-responsive and -non-responsive groups using Fisher’s exact test (Table 3). Before adjustment for multiple comparisons, *fnbA* and *sei* showed nominal differences in prevalence between the two groups (raw *p* = 0.039 and 0.011, respectively). However, these associations did not remain statistically significant after Benjamini–Hochberg false discovery rate correction (*fnbA*, adjusted *p* = 0.156; *sei*, adjusted *p* = 0.088). No virulence genes showed statistically significant differences between the two groups after FDR adjustment.

### 2.5. Association Between VF Gene Status and CCCP-Mediated MIC Reduction

**Figure 3:**
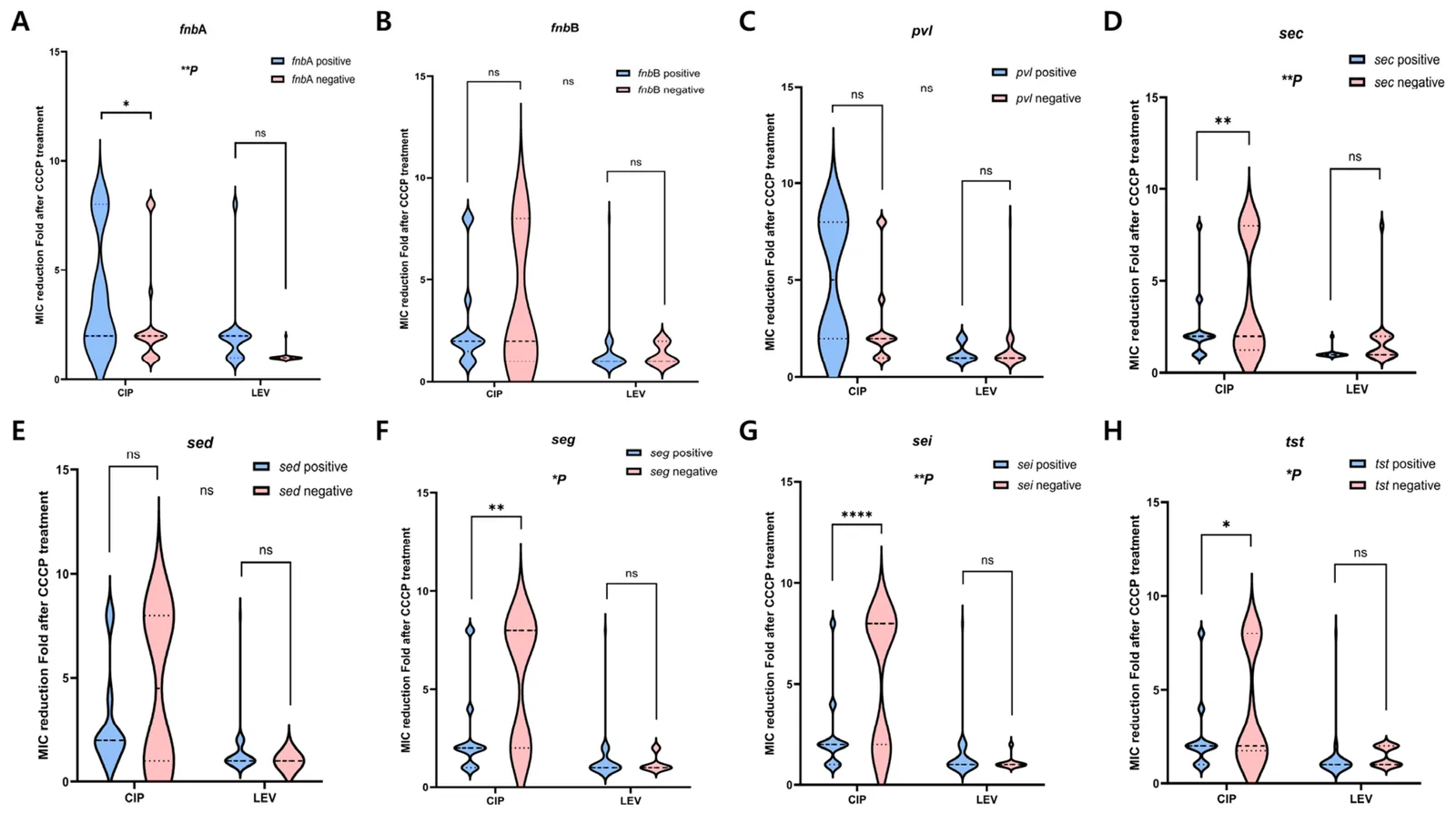
Comparison of CCCP-mediated MIC fold reductions according to virulence gene status. CCCP-mediated MIC reduction was calculated as the ratio of MIC in the absence of CCCP to that in the presence of CCCP for ciprofloxacin (CIP) and levofloxacin (LEV). MIC fold reductions were compared between virulence gene-positive and -negative MRSA isolates for (**A**) *fnbA*, (**B**) *fnbB*, (**C**) *pvl*, (**D**) *sec*, (**E**) *sed*, (**F**) *seg*, (**G**) *sei*, and (**H**) *tst*. Overall differences according to virulence gene status were assessed using two-way repeated-measures ANOVA, followed by Šídák’s multiple-comparisons test for comparisons between virulence gene-positive and -negative isolates for each antibiotic. The *p*-value shown above each panel represents the overall effect of virulence gene status from the two-way repeated-measures ANOVA. Virulence gene categories were as follows: adhesion (*fnbA*, *fnbB*), other toxin-associated (*pvl*), and superantigen (*sec*, *sed*, *seg*, *sei*, *tst*). * *p* < 0.05, ** *p* < 0.01 and **** *p* < 0.0001; ns, not significant.

CCCP-mediated MIC reduction was compared between virulence gene-positive and -negative isolates to evaluate associations between VF profiles and CCCP responsiveness (Figure 3). Significant overall effects of virulence gene status on CCCP-mediated MIC reduction were observed for *fnbA* (*p* = 0.0061), *sec* (*p* = 0.0035), *seg* (*p* = 0.0332), *sei* (*p* = 0.0030), and *tst* (*p* = 0.0462), whereas no significant overall effects were observed for *fnbB*, *pvl*, or *sed* (Figure 3). In the Šídák-adjusted multiple comparisons for each antimicrobial agent, *fnbA*-positive isolates exhibited significantly greater MIC fold reductions than *fnbA*-negative isolates for CIP (*p* = 0.0374), whereas no significant difference was observed for LEV (*p* = 0.1110). In contrast, *sec*-positive isolates showed significantly lower MIC fold reductions than *sec*-negative isolates for CIP (*p* = 0.0035), with no significant difference for LEV (*p* = 0.3029). Similarly, *seg*- and *sei*-positive isolates exhibited significantly lower CIP MIC fold reductions than their respective gene-negative isolates (*p* = 0.0011 and *p* < 0.0001, respectively), whereas no significant differences were observed for LEV (*p* = 0.9889 and *p* = 0.8955, respectively). *tst*-positive isolates also exhibited significantly lower CIP MIC fold reductions than *tst*-negative isolates (*p* = 0.0140), with no significant difference for LEV (*p* = 0.9003). No significant individual differences according to *fnbB*, *pvl*, or *sed* status were observed for either antimicrobial agent.

### 2.6. Association Between VF Gene Status and QRDR Mutation Numbers

**Figure 4:**
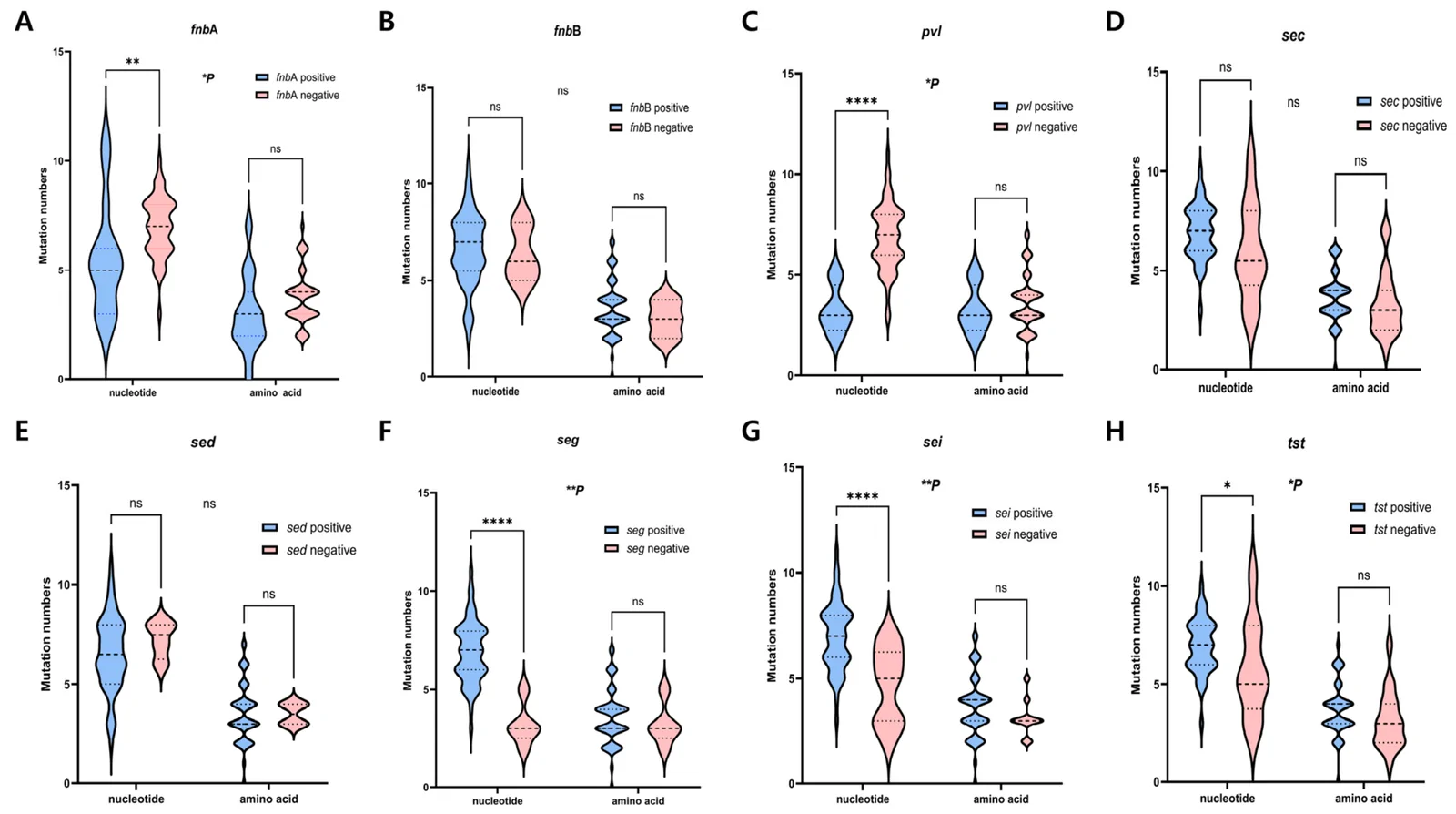
Comparison of QRDR mutation numbers according to virulence gene status. QRDR mutation numbers were evaluated based on the numbers of nucleotide mutations and amino acid substitutions identified in the QRDRs of *gyr*A, *gyr*B, *par*C, and *par*E. Mutation numbers were compared between virulence gene-positive and -negative MRSA isolates for (**A**) *fnb*A, (**B**) *fnb*B, (**C**) *pvl*, (**D**) *sec*, (**E**) *sed*, (**F**) *seg*, (**G**) *sei*, and (**H**) *tst*. Overall differences according to virulence gene status were assessed using two-way repeated-measures ANOVA, followed by Šídák’s multiple-comparisons test for comparisons between virulence gene-positive and -negative isolates at the nucleotide and amino acid levels. The *p*-value shown above each panel represents the overall effect of virulence gene status from the two-way repeated-measures ANOVA. Virulence gene categories were as follows: adhesion (*fnb*A, *fnb*B), other toxin-associated (*pvl*), and superantigen (*sec*, *sed*, *seg*, *sei*, *tst*). * *p* < 0.05, ** *p* < 0.01 and **** *p* < 0.0001; ns, not significant.

QRDR mutation numbers were compared between virulence gene-positive and -negative MRSA isolates at the nucleotide and amino acid levels (Figure 4). Significant overall effects of virulence gene status on QRDR mutation numbers were observed for *fnbA* (*p* = 0.0107), *pvl* (*p* = 0.0139), *seg* (*p* = 0.0040), *sei* (*p* = 0.0078), and *tst* (*p* = 0.0486), whereas no significant overall effects were observed for *fnbB*, *sec*, or *sed* (Figure 4).

In the Šídák-adjusted multiple comparisons, *fnbA*-positive isolates exhibited significantly fewer nucleotide mutations than *fnbA*-negative isolates (*p* = 0.0034), whereas no significant difference was observed in the number of amino acid substitutions (*p* = 0.1586). Similarly, *pvl*-positive isolates showed significantly fewer nucleotide mutations than *pvl*-negative isolates (*p* < 0.0001), with no significant difference at the amino acid level (*p* = 0.9015). In contrast, *seg*- and *sei*-positive isolates exhibited significantly greater numbers of nucleotide mutations than their respective gene-negative isolates (both *p* < 0.0001), whereas no significant differences were observed in the numbers of amino acid substitutions (*p* = 0.8272 and *p* = 0.5292, respectively). *tst*-positive isolates also showed significantly more nucleotide mutations than *tst*-negative isolates (*p* = 0.0214), with no significant difference at the amino acid level (*p* = 0.4060). No significant individual differences at either the nucleotide or amino acid level were observed according to *fnbB*, *sec*, or *sed* status.

Taken together, these results revealed contrasting patterns according to the functional categories of virulence genes. Among the adhesion-associated genes, *fnbA*-positive isolates showed fewer QRDR nucleotide mutations but greater CCCP-mediated CIP MIC reductions. In contrast, among the superantigen-associated genes, *seg*-, *sei*-, and *tst*-positive isolates showed greater numbers of QRDR nucleotide mutations and lower CCCP-mediated CIP MIC reductions. These findings suggest contrasting associations between *fnbA* and the superantigen-associated genes *seg*, *sei*, and *tst* across the two analyses.

## 3. Discussion

Fluoroquinolone resistance in *S. aureus* is a multifactorial phenotype involving target site alterations and efflux pump-mediated resistance mechanisms [22]. Mutations in the QRDRs of DNA gyrase and topoisomerase IV are major determinants of fluoroquinolone resistance, whereas multidrug efflux pump systems can further contribute to resistance by reducing intracellular antimicrobial concentrations [8]. In addition to antimicrobial resistance, *S. aureus* possesses a diverse repertoire of VFs involved in adhesion, host cell damage, immune evasion, and toxin-mediated pathogenicity [23]. However, the extent to which different fluoroquinolone resistance mechanisms are associated with distinct virulence profiles remains unclear.

In the present study, we investigated the relative contributions of QRDR mutations and CCCP-responsive mechanisms to fluoroquinolone resistance in clinical MRSA isolates and examined whether these resistance phenotypes are associated with distinct virulence gene profiles. The principal findings were that CCCP treatment significantly reduced both CIP and LEV MICs (Figure 1), although changes in LEV MICs were limited to a subset of isolates, and that isolates exhibiting a ≥4-fold reduction in CIP MICs carried fewer QRDR mutations than CCCP-non-responsive isolates. Furthermore, virulence-associated gene profiles showed suggestive differences between CCCP-responsive and -non-responsive isolates, with adhesion-associated factors, *fnb*A, tending to occur more frequently in the CCCP-responsive phenotype, whereas several superantigen genes, *seg*, *sei*, and *tst*, tended to be associated with the CCCP-non-responsive, higher-mutation phenotype (Figure 3 and Figure 4). Together, these observations suggest heterogeneous associations between QRDR mutation burden and CCCP responsiveness in fluoroquinolone-resistant MRSA isolates and indicate that these phenotypes coexist with distinct virulence gene profiles.

Efflux pump-mediated resistance is an important complementary mechanism for target site modification in *S. aureus*. Multidrug efflux pumps, particularly members of the Nor family, can reduce the intracellular concentrations of structurally diverse antimicrobial compounds, including fluoroquinolones. NorA is well characterized for its ability to extrude hydrophilic fluoroquinolones, such as CIP, and the inhibition of efflux pump activity can consequently increase intracellular antimicrobial concentrations and restore susceptibility [24]. In the present study, CCCP treatment significantly reduced both CIP and LEV MICs. The reduction was more broadly apparent for CIP, whereas LEV MICs remained unchanged in 48 of the 60 isolates, indicating that the statistically significant LEV response was limited to a subset of isolates (Figure 1). These differential response patterns suggest that CCCP-sensitive mechanisms may contribute differently to CIP and LEV susceptibility, with a more pronounced overall response to CCCP observed for CIP. However, CCCP-mediated changes in antimicrobial susceptibility are not uniformly characterized by MIC reduction. A previous study reported that CCCP reduces antimicrobial MICs in several *Acinetobacter baumannii* isolates, whereas some isolates paradoxically exhibit increased MICs following CCCP exposure, a phenomenon discussed in relation to the Eagle effect [25]. By contrast, CIP or LEV MICs did not increase following CCCP treatment in the MRSA isolates in the present study (Table 1).

Notably, the degree of CCCP response was associated with the number of QRDR mutations. Isolates exhibiting a ≥4-fold reduction in CIP MIC following CCCP treatment generally carried fewer QRDR mutations, whereas CCCP-non-responsive isolates exhibited more mutations (Figure 2). Fluoroquinolone resistance in *S. aureus* is classically mediated by mutations in the QRDRs of DNA gyrase and topoisomerase IV, whereas efflux pump systems provide an additional mechanism by decreasing intracellular drug exposure [7]. Among the individual QRDR targets, the differences between the two groups were particularly pronounced for *gyr*A, with the CCCP-non-responsive group exhibiting significantly more *gyr*A nucleotide mutations and GyrA amino acid substitutions (Figure 2). This finding suggests that *gyr*A alterations contribute substantially to the greater number of QRDR mutations observed in isolates that show limited responsiveness to CCCP treatment. Interestingly, this pattern differs from our previous findings, in which the number of *par*C mutations was significantly associated with fluoroquinolone MIC levels and specific ParC substitutions were associated with higher levels of resistance [4]. Therefore, the present findings suggest that different QRDR components are associated with distinct aspects of fluoroquinolone resistance. In specific, *par*C alterations might be more closely related to the magnitude of fluoroquinolone resistance, whereas *gyr*A alterations might be more prominent in distinguishing isolates according to their responsiveness to CCCP treatment. These observations further support the multifactorial nature of fluoroquinolone resistance in MRSA, in which target site mutations and CCCP-responsive mechanisms may contribute differently to the overall resistance phenotype.

Our findings extend this concept by suggesting an inverse relationship between the apparent contribution of CCCP-responsive mechanisms and QRDR mutation number. Thus, isolates carrying fewer target site mutations tended to exhibit greater CCCP-mediated reductions in fluoroquinolone MIC, whereas isolates with accumulated QRDR alterations showed comparatively lower responsiveness to CCCP treatment, suggesting an inverse association between QRDR mutation numbers and CCCP responsiveness.

An additional finding of this study was the association between resistance phenotypes and virulence gene profiles. The distribution of virulence genes according to CCCP responsiveness was broadly consistent with the patterns observed in the analyses of CCCP-mediated MIC reduction and QRDR mutation numbers according to virulence gene status (Table 3, Figure 3 and Figure 4). Among the virulence determinants examined, *fnbA* showed a nominal association with the CCCP-responsive phenotype. The prevalence of *fnbA* was higher in the CCCP-responsive group than in the CCCP-non-responsive group (46.7% vs. 17.8%; raw *p* = 0.039), although this association did not remain statistically significant after FDR correction (adjusted *p* = 0.156; Table 3). Consistent with this pattern, *fnbA*-positive isolates exhibited greater CCCP-mediated MIC reductions, particularly for CIP (Figure 3), and significantly fewer QRDR nucleotide mutations than *fnbA*-negative isolates (Figure 4). Taken together, these complementary findings suggest a potential association between *fnbA* and a CCCP-responsive phenotype characterized by relatively greater CCCP-mediated MIC reduction and fewer QRDR mutations.

Fibronectin-binding proteins are major surface-associated adhesins of *S. aureus* that mediate attachment to extracellular matrix components, facilitate host cell invasion, and contribute to biofilm-associated phenotypes [26]. Interestingly, efflux pump systems are increasingly recognized to function beyond antimicrobial resistance. In *S. aureus*, efflux pump activity has been linked to biofilm formation and bacterial adaptation, suggesting that the mechanisms controlling antimicrobial extrusion intersect with the phenotypes involved in persistence and colonization [20]. Although the present study did not directly assess biofilm formation or *fnbA* expression, the enrichment of this adhesion-associated gene among the CCCP-responsive isolates raises the possibility that CCCP-responsive phenotypes, potentially involving efflux-related mechanisms, may coexist with bacterial traits that favor adhesion or persistence.

By contrast, several superantigen-associated genes showed an overall tendency toward the CCCP-non-responsive, higher-mutation phenotype. Among these genes, *sei* showed the strongest nominal association in the categorical analysis. The prevalence of *sei* was higher in the CCCP-non-responsive group than in the CCCP-responsive group (91.1% vs. 60.0%; raw *p* = 0.011), although this association did not remain statistically significant after FDR correction (adjusted *p* = 0.088; Table 3). Consistent with this tendency, *sei*-positive isolates exhibited significantly lower CCCP-mediated CIP MIC reductions and significantly greater numbers of QRDR nucleotide mutations than *sei*-negative isolates (Figure 3 and Figure 4). Similar patterns were observed for *seg* and *tst*, for which gene-positive isolates showed lower CCCP-mediated CIP MIC reductions together with greater numbers of QRDR nucleotide mutations. These genes were more prevalent in the CCCP-non-responsive group, although the differences in prevalence were not statistically significant after FDR correction. Moreover, *sec* was associated with lower CCCP-mediated MIC reductions, although this pattern was not accompanied by a significant difference in the number of QRDR mutations (Figure 3 and Figure 4). Together, these findings suggest broadly consistent patterns across the categorical and continuous analyses. The relationship between antimicrobial resistance and virulence in *S. aureus* is complex and does not necessarily follow a simple positive or negative correlation. The strongest concordant patterns across Figure 3 and Figure 4 were therefore observed for *fnbA*, *seg*, *sei*, and *tst*, with *fnbA* showing greater CCCP-mediated CIP reduction and fewer nucleotide mutations, and the superantigen-associated genes *seg*, *sei*, and *tst* showing the opposite pattern. The present analysis evaluated the presence or absence of genes rather than their expression or toxin production; thus, these findings should be interpreted as associations between genetic profiles rather than evidence of enhanced virulence activity.

This study has some limitations that should be considered when interpreting the findings. First, CCCP is a protonophore that disrupts the proton motive force and is not specific to a single efflux pump. Therefore, CCCP-associated MIC reductions provide indirect evidence of efflux pump involvement but cannot identify the specific transporter responsible. Although NorA and other multidrug efflux pump systems contribute to antimicrobial resistance in *S. aureus*, multiple efflux pump genes, including *nor*A, *nor*B, *nor*C, *mep*A, and *mde*A, have been reported in clinical isolates. Second, VFs were evaluated at the gene level, and the presence of genes did not necessarily reflect transcription, protein production, or functional virulence.

Therefore, future studies incorporating quantitative expression analyses of efflux pump genes, functional efflux pump assays, virulence gene expression, and larger collections of genetically diverse clinical isolates are necessary to clarify the molecular basis of these associations. Further characterization of the relationships among QRDR mutations, CCCP-responsive phenotypes, functionally validated efflux mechanisms, and virulence profiles may provide a basis for the development of resistance mechanism-oriented diagnostic approaches. If specific molecular signatures could reliably distinguish isolates in which target site mutations or functionally validated efflux mechanisms predominantly contribute to fluoroquinolone resistance, the combined detection of QRDR mutations, efflux pump-associated markers, and virulence genes might facilitate a more detailed characterization of resistant MRSA isolates. Furthermore, the identification of a functionally validated efflux-associated phenotype may have therapeutic implications for the future development of safe and selective efflux pump inhibitors as antibiotic adjuvants. However, CCCP itself is not suitable for therapeutic use because of its nonspecific disruption of the proton motive force and associated toxicity. Therefore, larger clinical studies and functional validations of individual efflux pump systems are required before these findings can be translated into diagnostic or therapeutic applications.

This integrated characterization may improve our understanding of the biological diversity underlying fluoroquinolone-resistant MRSA and provide a basis for future studies investigating the interplay between antimicrobial resistance and virulence.

## 4. Materials and Methods

### 4.1. Study Design

**Figure 5:**
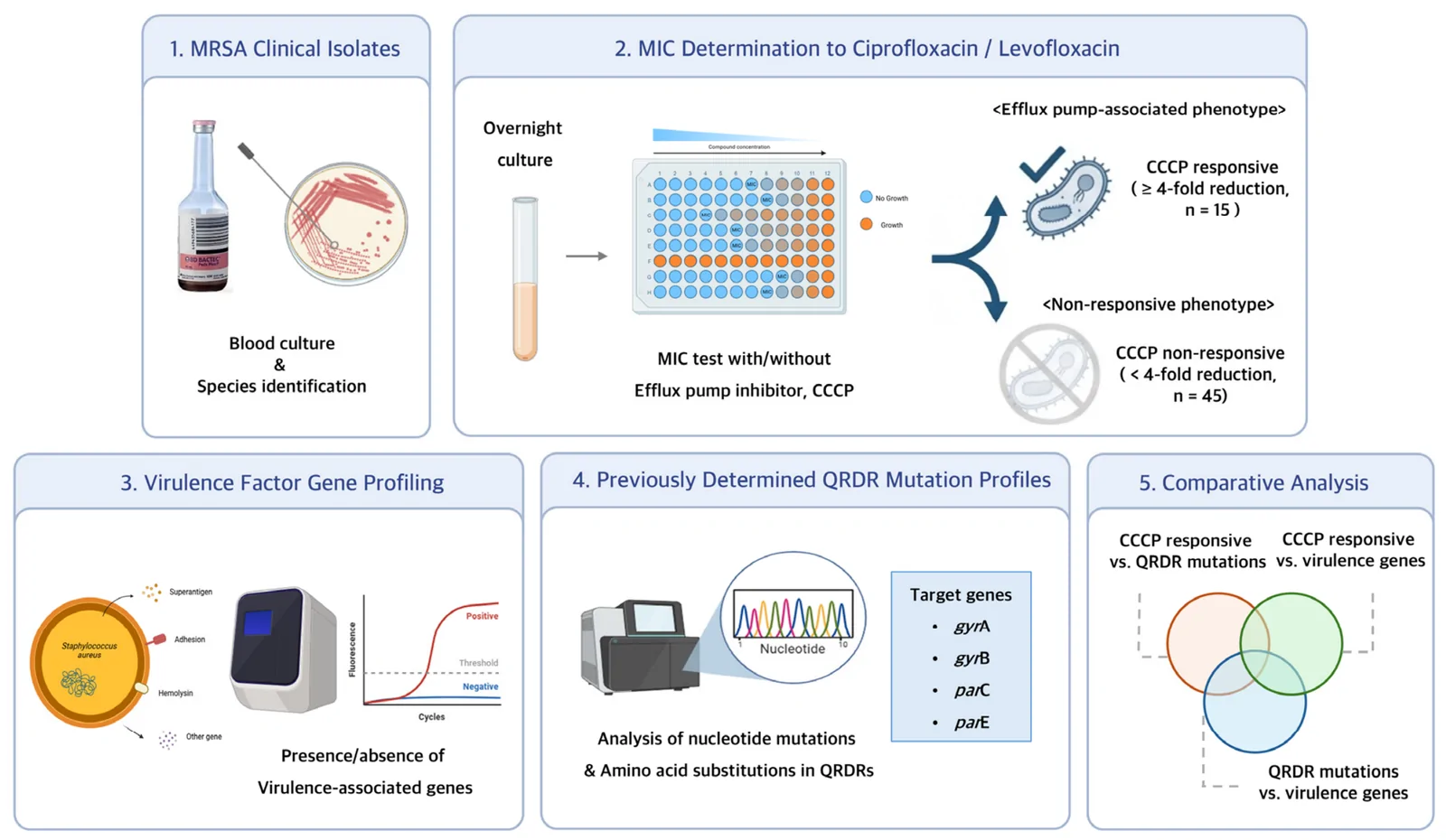
Schematic of the experimental design and analytical workflow.

The overall experimental design and analytical workflow of this study are shown in Figure 5. Briefly, 60 fluoroquinolone-resistant MRSA bloodstream isolates were evaluated for ciprofloxacin (CIP) and levofloxacin (LEV) susceptibility in the presence and absence of carbonyl cyanide 3-chlorophenylhydrazone (CCCP). The isolates were classified according to CCCP-mediated changes in CIP MICs, and these phenotypic responses were compared with previously characterized quinolone resistance-determining region (QRDR) mutation profiles and virulence-associated gene profiles.

### 4.2. Bacterial Isolates

A total of 60 fluoroquinolone-resistant MRSA clinical isolates were used in this study. These isolates constituted the MRSA subset of 63 fluoroquinolone-resistant *S. aureus* bloodstream isolates (60 MRSA and 3 MSSA) previously characterized in our study. The isolates were collected and identified as described previously [4].

### 4.3. Genomic DNA Extraction

Genomic DNA (gDNA) was extracted from single colonies of the cultured *S. aureus* isolates using a 5% Chelex Resin-based method. Briefly, a single colony was suspended in 500 μL of 5% Chelex Resin solution (Bio-Rad, Hercules, CA, USA), boiled for 10 min, and centrifuged at 3000× *g* for 10 min. The supernatant was transferred to a sterile tube, and the concentration and purity of the extracted gDNA were assessed using a NanoDrop 2000 spectrophotometer (Thermo Fisher Scientific, Waltham, MA, USA). Extracted gDNA was stored at −20 °C until further molecular analysis.

### 4.4. QRDR Mutation Profiles

The QRDR mutation profiles of *gyr*A, *gyr*B, *par*C, and *par*E in the 60 MRSA isolates were obtained from our previous study [4]. Briefly, the QRDRs of these genes were amplified by conventional PCR, and the PCR products were confirmed by agarose gel electrophoresis and subjected to Sanger sequencing. The resulting nucleotide sequences were aligned with the corresponding sequences of *S. aureus* ATCC 12600 to identify nucleotide mutations, and the sequences were translated to determine the corresponding amino acid substitutions. These mutation profiles were used in the present study to evaluate their associations with CCCP responsiveness and CCCP-mediated changes in fluoroquinolone susceptibility.

### 4.5. Assessment of CCCP Effects on Bacterial Growth

Before performing the efflux pump inhibition assay, the effect of CCCP (C2759, Sigma-Aldrich, St. Louis, MO, USA) on bacterial growth was evaluated using the broth microdilution method to determine an appropriate working concentration. CCCP was dissolved in dimethyl sulfoxide (DMSO) and diluted in Mueller–Hinton (MH) broth to the desired concentrations. To exclude a potential solvent-related effect, the influence of DMSO on bacterial growth was evaluated separately using seven *S. aureus* strains. DMSO was tested by two-fold serial dilution from 5% to 0.039% (*v*/*v*), and bacterial growth was assessed by measuring optical density at 595 nm relative to the corresponding DMSO-free growth controls in MH broth. No substantial growth suppression was observed across the tested DMSO concentrations. Following an initial concentration screening, CCCP concentrations of 0.1, 0.15, 0.18, and 0.2 μg/mL were further evaluated using eight *S. aureus* strains, including three MRSA clinical isolates, two NCCP reference strains, and three MSSA strains. Bacterial growth in the presence and absence of CCCP was assessed by measuring the optical density at 600 nm (OD_600_) using an iMARK Microplate Reader (Bio-Rad, Hercules, CA, USA). Relative OD_600_ values were calculated as percentages of the corresponding untreated growth controls. A 50% relative OD_600_ level was used as an operational screening threshold to identify concentrations associated with substantial direct growth suppression. This threshold was based solely on OD600 measurements and was not intended to represent a 50% reduction in viable bacterial counts (CFU).

CCCP was prepared as a 20 mg/mL stock solution in DMSO. At the final CCCP concentration of 0.1 μg/mL selected for subsequent experiments, the corresponding final DMSO concentration was approximately 0.0005% (*v*/*v*). Across the CCCP screening range of 0.1–0.2 μg/mL, the final DMSO concentrations were approximately 0.0005–0.001% (*v*/*v*), substantially below the concentrations evaluated in the DMSO control experiment.

Based on the overall OD_600_ responses across the tested strains and concentrations, 0.1 μg/mL CCCP was selected as the working concentration for subsequent efflux inhibition and antimicrobial susceptibility experiments.

### 4.6. Determination of Fluoroquinolone MICs in the Presence of CCCP

The MICs of CIP and LEV were determined in the presence and absence of CCCP using the broth microdilution method. CIP (≥98% purity, catalog no. 17850; Sigma-Aldrich) was dissolved in 0.1 N HCl. LEV (98.0–102.0% anhydrous basis, catalog no. 28266; Sigma-Aldrich) and carbonyl cyanide 3-chlorophenylhydrazone (CCCP; ≥97% purity, catalog no. C2759; Sigma-Aldrich) were dissolved in DMSO. The prepared stock solutions were subsequently diluted to the concentrations required for the experiments. Each MRSA isolate was cultured in MH broth, and the bacterial suspension was adjusted to a 0.5 McFarland standard using a DensiCHEK Plus instrument (bioMérieux, Marcy-l’Étoile, France). The suspension was diluted in MH broth to prepare the inoculum.

CIP and LEV were prepared using two-fold serial dilutions in 96-well microplates to obtain final concentrations ranging from 0.5 μg/mL to 512 μg/mL. For CCCP treatment, CCCP was added to each well at a final concentration of 0.1 μg/mL. An equivalent assay without CCCP was performed as an untreated control. The bacterial suspension was added to each well to obtain a final inoculum concentration of 5 × 10^5^ CFU/mL in a total volume of 200 μL. A growth control well containing a bacterial suspension without antibiotics was also included.

The microplates were incubated at 37 °C for 18 h. Following incubation, bacterial growth was evaluated by measuring the optical density at 595 nm using an iMARK Microplate Reader (Bio-Rad). The MICs of CIP and LEV obtained in the presence of CCCP were compared with those obtained in its absence to evaluate CCCP-mediated changes in fluoroquinolone susceptibility.

### 4.7. Classification of MRSA Isolates According to CCCP-Mediated MIC Reduction

The effect of CCCP on fluoroquinolone susceptibility was expressed as a fold reduction in MIC, calculated as the ratio of the MIC obtained in the absence of CCCP to that obtained in the presence of CCCP. Fold reductions were calculated separately for CIP and LEV.

For phenotypic classification, the fold reduction in CIP MIC was used. Based on a previously established criterion, isolates exhibiting a ≥4-fold reduction in CIP MIC in the presence of CCCP were classified as CCCP responsive, whereas those exhibiting a <4-fold reduction were classified as CCCP non-responsive [25].

### 4.8. Analysis of QRDR Mutation Number According to CCCP Responsiveness

The QRDR mutation profiles of *gyr*A, *gyr*B, *par*C, and *par*E in the 60 MRSA isolates were determined by sequence analysis as described in a previous study [4]. These previously characterized mutation data were reanalyzed according to CCCP responsiveness.

For each isolate, the number of mutations was determined based on the number of mutations identified within the QRDRs of *gyr*A, *gyr*B, *par*C, and *par*E. Mutation numbers were analyzed at the nucleotide and amino acid levels. The number of mutations in each QRDR gene and the total number of nucleotide mutations and amino acid substitutions were compared between the CCCP-responsive and CCCP-non-responsive groups.

### 4.9. Design of Primer Pairs for 21 S. aureus Virulence Factor Genes

A total of 21 virulence-associated genes were examined, including adhesion genes (*clf*A, *clf*B, *fnb*A, and *fnb*B), hemolysin genes (*hla*, *hlb*, *hlg*, and *hld*), superantigen genes (*sea*, *seb*, *sec*, *sed*, *see*, *seg*, *seh*, *sei*, and *tst*), and other toxin-associated genes (*pvl*, *lukED*, *eta*, and *etb*). Primer sequences for eight staphylococcal enterotoxin genes (*sea*, *seb*, *sec*, *sed*, *see*, *seg*, *seh*, and *sei*) were obtained from a previously published study [27]. Primer pairs for the remaining 13 virulence-associated genes were designed using sequences available in the National Center for Biotechnology Information GenBank database and Primer3 software. Synthetic positive controls corresponding to the target virulence genes were used for assay validation. The characteristics and sequences of the primer pairs are listed in Table 4.

### 4.10. Detection of S. aureus Virulence Factor Genes Using qPCR SYBR Green Assay

The qPCR SYBR Green assay was conducted to detect virulence gene patterns. In brief, 10 μL of THUNDERBIRD SYBR qPCR Mix (Toyobo, Osaka, Japan), 10 pmol of each forward and reverse primer, 3 μL of extracted gDNA as template DNA, and 5 μL of sterile distilled water were added to prepare a 20 μL mixture. A qPCR was performed using the QuantSTUDIO 7 Flex Real-Time PCR Instrument (Thermo Fisher Scientific, Waltham, MA, USA). The qPCR conditions were as follows: pre-denaturation at 94 °C for 10 min; 40 cycles of denaturation at 94 °C for 30 s, primer annealing at 55 °C for 30 s, and extension at 72 °C for 30 s; and a final extension at 72 °C for 10 min. After amplification, a melting curve was derived by increasing the temperature of the amplification product by increments of 0.5 °C, from 55 °C to 95 °C. The qPCR results were considered valid only when the Ct value of the amplification curve derived from the qPCR was less than 35. Synthetic DNA corresponding to the target virulence genes was included as a positive amplification control, and sterile distilled water was included as a no-template negative control in each qPCR assay.

### 4.11. Statistical Analysis

Statistical analyses were performed using GraphPad Prism version 10.1.2 (GraphPad Software, Boston, MA, USA). The MICs of CIP and LEV before and after CCCP treatment were compared using the Wilcoxon matched-pairs signed-rank test. Differences in QRDR mutation numbers according to CCCP responsiveness were assessed using two-way repeated-measures analysis of variance (ANOVA), with individual mutation categories and total mutation numbers further compared using unpaired *t*-tests. Associations of virulence gene status with CCCP-mediated MIC reduction and QRDR mutation numbers were analyzed using two-way repeated-measures ANOVA, followed by Šídák’s multiple-comparisons test for individual comparisons. Differences in the prevalence of virulence genes between the CCCP-responsive and CCCP-non-responsive groups were assessed using Fisher’s exact test, and the resulting *p*-values were adjusted for multiple comparisons using the Benjamini–Hochberg false discovery rate (FDR) method. Statistical significance was considered at *p* < 0.05.

## 5. Conclusions

QRDR mutations and CCCP-responsive mechanisms exert heterogeneous contributions to fluoroquinolone resistance in MRSA clinical isolates. Treatment with the efflux pump inhibitor CCCP significantly reduced both CIP and LEV MICs, although changes in LEV MICs were limited to a subset of isolates. Isolates exhibiting a CCCP-responsive phenotype carried fewer QRDR mutations, whereas CCCP-non-responsive isolates showed more mutations, suggesting differences in the relative contributions of CCCP-sensitive and target mutation-associated resistance mechanisms. Furthermore, these resistance phenotypes were associated with distinct virulence gene profiles. *fnbA*-positive isolates showed greater CCCP-mediated CIP MIC reduction and fewer QRDR nucleotide mutations, whereas isolates positive for the superantigen-associated genes *seg*, *sei*, and *tst* showed lower CCCP-mediated CIP MIC reductions and greater numbers of QRDR nucleotide mutations. These findings highlight a potential relationship between fluoroquinolone resistance mechanisms and the virulence genetic background of MRSA. Further functional and molecular studies are required to elucidate the mechanisms underlying these associations and their potential biological and clinical significance.

## Figures and Tables

**Table 1:** MICs of ciprofloxacin and levofloxacin against MRSA isolates in the absence and presence of CCCP.

Isolate	Ciprofloxacin MIC (µg/mL)		Levofloxacin MIC (µg/mL)	
	Without CCCP	With CCCP	Without CCCP	With CCCP
1	128	64	16	16
2	32	8	4	4
3	128	64	16	16
4	32	16	8	4
5	128	64	16	16
6	32	16	4	4
7	32	16	4	4
8	512	256	512	512
9	512	512	512	512
10	64	64	512	512
11	128	64	16	16
12	512	512	512	512
13	16	8	4	4
14	128	64	16	16
15	16	16	4	4
16	64	64	128	128
17	128	64	128	128
18	64	64	64	64
19	128	64	16	16
20	16	16	4	4
21	128	64	16	16
22	128	64	128	128
23	128	64	16	16
24	128	64	16	16
25	512	256	512	512
26	16	8	4	2
27	32	32	8	4
28	64	64	128	128
29	128	64	16	16
30	128	64	16	16
31	128	64	16	16
32	16	8	4	2
33	64	64	128	128
34	64	64	16	16
35	256	256	512	512
36	16	8	4	2
37	64	64	16	16
38	256	256	32	32
39	128	64	16	16
40	128	64	16	16
41	32	16	8	4
42	16	8	4	4
43	64	32	256	256
44	2	0.5	1	0.5
45	256	256	32	32
46	128	64	128	128
47	64	16	256	128
48	16	8	8	4
49	32	4	4	4
50	256	32	16	16
51	64	8	4	4
52	128	16	8	8
53	256	32	16	8
54	64	8	8	4
55	1024	128	32	32
56	256	64	128	128
57	1024	128	32	32
58	512	64	16	16
59	256	64	16	16
60	32	4	16	2

**Table 2:** Distribution of virulence factor genes showing variable prevalence among *S. aureus* isolates.

Group	Virulence Factor	Positive, *n* (%)	Negative, *n* (%)	Total (%)
Adhesion genes	*fnb*A	15 (25.0%)	45 (75.0%)	60 (100)
	*fnb*B	57 (95.0%)	3 (5.0%)	60 (100)
Superantigens	*sec*	40 (66.7%)	20 (33.3%)	60 (100)
	*sed*	4 (6.7%)	56 (93.3%)	60 (100)
	*seg*	55 (91.7%)	5 (8.3%)	60 (100)
	*sei*	50 (83.3%)	10 (16.7%)	60 (100)
	*tst*	42 (70.0%)	18 (30.0%)	60 (100)
Others	*pvl*	4 (6.7%)	56 (93.3%)	60 (100)

Only virulence factor genes showing variable presence among the isolates are shown. Genes that were uniformly positive (*clf*A, *clf*B, *hla*, *hlb*, *hld*, and *lukED*) or uniformly negative (*hlg*, *sea*, *seb*, *see*, *seh*, *eta*, and *etb*) were excluded from the comparative analyses.

**Table 3:** Distribution of virulence factor genes according to CCCP responsiveness among MRSA isolates.

Group	Virulence Factor	CCCP-Responsive Group (*n* = 15), Positive *n* (%)	CCCP-Non-Responsive Group (*n* = 45), Positive *n* (%)	Raw *p*-Value	FDR-Adjusted *p*-Value
Adhesion genes	*fnb*A	7 (46.7%)	8 (17.8%)	0.039	0.156
	*fnb*B	14 (93.3%)	43 (95.6%)	1.000	1.000
Superantigens	*sec*	7 (46.7%)	33 (73.3%)	0.111	0.189
	*sed*	2 (13.3%)	2 (4.4%)	0.258	0.295
	*seg*	12 (80.0%)	43 (95.6%)	0.094	0.189
	*sei*	9 (60.0%)	41 (91.1%)	0.011	0.088
	*tst*	8 (53.3%)	34 (75.6%)	0.118	0.189
Others	*pvl*	2 (13.3%)	2 (4.4%)	0.258	0.295

Differences between the CCCP-responsive and CCCP-non-responsive groups were analyzed using Fisher’s exact test. Raw *p*-values were adjusted for multiple comparisons using the Benjamini–Hochberg false discovery rate (FDR) method. An FDR-adjusted *p*-value < 0.05 was considered statistically significant.

**Table 4:** Primer sequences and reference information for virulence-associated genes used in this study.

No.	Target Region		Primer Sequence (5’ to 3’)	Product Length (bp)	Gene Accession No.
	Adhesion genes				
1	Clumping factor A	F	GCC GGT AAC TGG TGA AGC T	313	MK001021
		R	TGC TCT CAT TCT AGG CGC AC		
2	Clumping factor B	F	CCG GTA GTA AAT GCT GCT G	101	HQ424298
		R	CTT TGA TTA GGG TCA AAT GTA GTC		
3	Fibronectin binding protein A	F	CAT TAT CCC AAG TTA AGG TAT ATC C	101	KP096552
		R	TGG TAC TGA TGA AGT TGA TTT TAG AA		
4	Fibronectin binding protein B	F	GGA GCG GCC TCA GTA TTC TT	201	X62992
		R	AGT TGA TGT CGC GCT GTA TG		
	Hemolysin genes				
5	α-hemolysin	F	CTG ATT ACT ATC CAA GAA ATT CGA TT	210	M90536
		R	CTT TCC AGC CTA CTT TTT TAT CAG T		
6	β-hemolysin	F	GTG CAC TTA CTG ACA ATA GTG C	310	KC242859
		R	GTT GAT GAG TAG CTA CCT TCA G		
7	γ-hemolysin	F	GTC ATA GAG TCC ATA ATG CAT TTA A	192	X81586
		R	TGT GAT TTT GCC TTC AGC ATT TG		
8	δ-hemolysin	F	CCC TCG CAA CTG ATA ATC CT	144	AF288215
		R	CAA CTG GGT CAT GCT TAC GA		
	Superantigens				
9	Enterotoxin A	F	GGT TAT CAA TGT GCG GGT GG	102	M18970
		R	CGG CAC TTT TTT CTC TTC GG		
10	Enterotoxin B	F	GTA TGG TGG TGT AAC TGA GC	164	M11118
		R	CCA AAT AGT GAC GAG TTA GG		
11	Enterotoxin C	F	CTT GTA TGT ATG GAG GAA TAA CAA	284	X05815
		R	TGC AGG CAT CAT ATC ATA CCA		
12	Enterotoxin D	F	GTG GTG AAA TAG ATA GGA CTG C	385	M28521
		R	TCA TAT GAA GGT GCT CTG TGG		
13	Enterotoxin E	F	GTA CCA ATT AAC TTG TGG ATA GAC	171	AY518387
		R	CCT CTT TGC ACC TTA CCG C		
14	Enterotoxin G	F	CAG GTA ACA ATC GAC AAT AGA CA	139	AB060535
		R	AGA ACC ATC AAA CTC GTA TAG C		
15	Enterotoxin H	F	AGG TGA TAG TGG CAA TGA TTT G	200	AB060536
		R	TAG CAC CAA TCA CCC TTT CC		
16	Enterotoxin I	F	CAA CTC GAA TTT TCA ACA GGT ACC	469	AB060537
		R	TTA CAG GCA GTC CAT CTC CTG		
17	Toxic shock syndrome toxin	F	ACC CCT GTT CCC TTA TCA TC	224	J02615
		R	CCT TTT GTA AAA GCA GGG CTA T		
	Others				
18	Panton-Valentine leukocidin	F	AAA TGC TGG ACA AAA CTT CTT GG	108	X72700
		R	TTT GCA GCG TTT TGT TTT CGA G		
19	Leukocidin ED	F	TGA AAA AGG TTC AAA GTT GAT ACG AG	268	Y13225
		R	GTA TTC GAT AGC AAA AGC AGT GC		
20	Exfoliative toxin A	F	ACT GTA GGA GCT AGT GCA TTT GT	189	M17347
		R	TGG ATA CTT TTG TCT ATC TTT TTC ATC		
21	Exfoliative toxin B	F	TAC AAG CAA AAG AAT ACA GCG C	233	M17348
		R	CGA TGG GTT TTT GGC TGC TTC		

Primer pairs for eight staphylococcal enterotoxin genes (*sea*, *seb*, *sec*, *sed*, *see*, *seg*, *seh*, and *sei*) were adopted from a previously published study [27].

## Data Availability

The original contributions presented in this study are included in the article material. Further inquiries can be directed to the corresponding authors.

## Abbreviations

The following abbreviations are used in this manuscript:

MRSA	Methicillin-resistant *Staphylococcus aureus*
QRDR	Quinolone resistance-determining region
MIC	Minimum inhibitory concentration
CCCP	Carbonyl cyanide 3-chlorophenylhydrazone
PVL	Panton–Valentine leucocidin
MSSA	Methicillin-susceptible *S. aureus*
CIP	Ciprofloxacin
VF	Virulence factor
DMSO	Dimethyl sulfoxide
MH	Mueller–Hinton
PCR	Polymerase chain reaction
qPCR	Quantitative real-time polymerase chain reaction
ANOVA	Analysis of variance
